# Supplementary material for: Asymmetric gene expression in grain development of reciprocal crosses between tetraploid and hexaploid wheats
Source: Commun Biol. 2022 Dec 23;5:1412. doi: 10.1038/s42003-022-04374-w (PMC9789062; doi:10.1038/s42003-022-04374-w)
Supplement: Supplementary file 2 — Supplementary Information [file 42003_2022_4374_MOESM2_ESM.pdf]

Supplementary Figures

Supplementary Figure 1 Grain development of reciprocal crosses between tetraploid and hexaploid wheats.

Supplementary Figure 2 Construction of a consensus transcriptome from multiple samples with different ploidy levels.

Supplementary Figure 3 Number of expressed genes detected at each stage (TPM>=1) in each sample.

Supplementary Figure 4 Principal component analysis (PCA) using TPM values of all genes from 144 samples.

Supplementary Figure 5 Number of differentially expressed genes (DEGs) during embryo development.

Supplementary Figure 6 Module eigengene values of clusters identified in AC Barrie x Commander (AxC).

Supplementary Figure 7 Module eigengene values of clusters identified in Commander x AC Barrie (CxA).

Supplementary Figure 8 Module eigengene values of clusters identified in AC Barrie x Strong Field (AxS).

Supplementary Figure 9 Module eigengene values of clusters identified in Strong Field x AC Barrie (SxA).

Supplementary Figure 10 Module eigengene values of clusters identified in Commander x Strong Field (CxS).

Supplementary Figure 11 Module eigengene values of clusters identified in Strong Field x Commander (SxC).

Supplementary Figure 12 Module eigengene values of clusters identified in AC Barrie x Chinese Spring (AxZ).

Supplementary Figure 13 Module eigengene values of clusters identified in Chinese Spring x AC Barrie (ZxA).

Supplementary Figure 14 Go enrichment analysis for clusters identified in different reciprocal crosses.

Supplementary Figure 15 Number of DEGs in embryo development essential pathways during embryo development.

Supplementary Figure 16 Number of DEGs shared between crosses.

Supplementary Figure 17 Go enrichment analysis for the up-regulated and down-regulated genes between reciprocal pairs in embryos.

Supplementary Figure 18 Normalized expression of differentially expressed genes between reciprocal crosses across all stages.

Supplementary Figure 19 Number of DEGs in embryo development essential pathways between reciprocal crosses.

Supplementary Figure 20 Summary of alternative splicing events identified in reciprocal crosses between hexploid and tetraploid wheats.

Supplementary Figure 21 Distribution of genes, alternative splicing (AS) events and homeologous triads on wheat chromosomes identified in all samples in this study.

Supplementary Figure 22 Synteny plot of homeologous triads on wheat chromosomes.

Supplementary Figure 23 Number of triads in each category acrossseven embryo stages.

Supplementary Figure 24 Distribution of percent values for each homeologous gene in different categories.

Supplementary Figure 25 Changes in triad classification between reciprocal crosses at each developmental stage.

Supplementary Figure 26 Number of changes in triad classification between reciprocal crosses.

Supplementary Figure 27 Scatter plots for imprinting genes.

Supplementary Figure 28 Identification of imprinting genes.

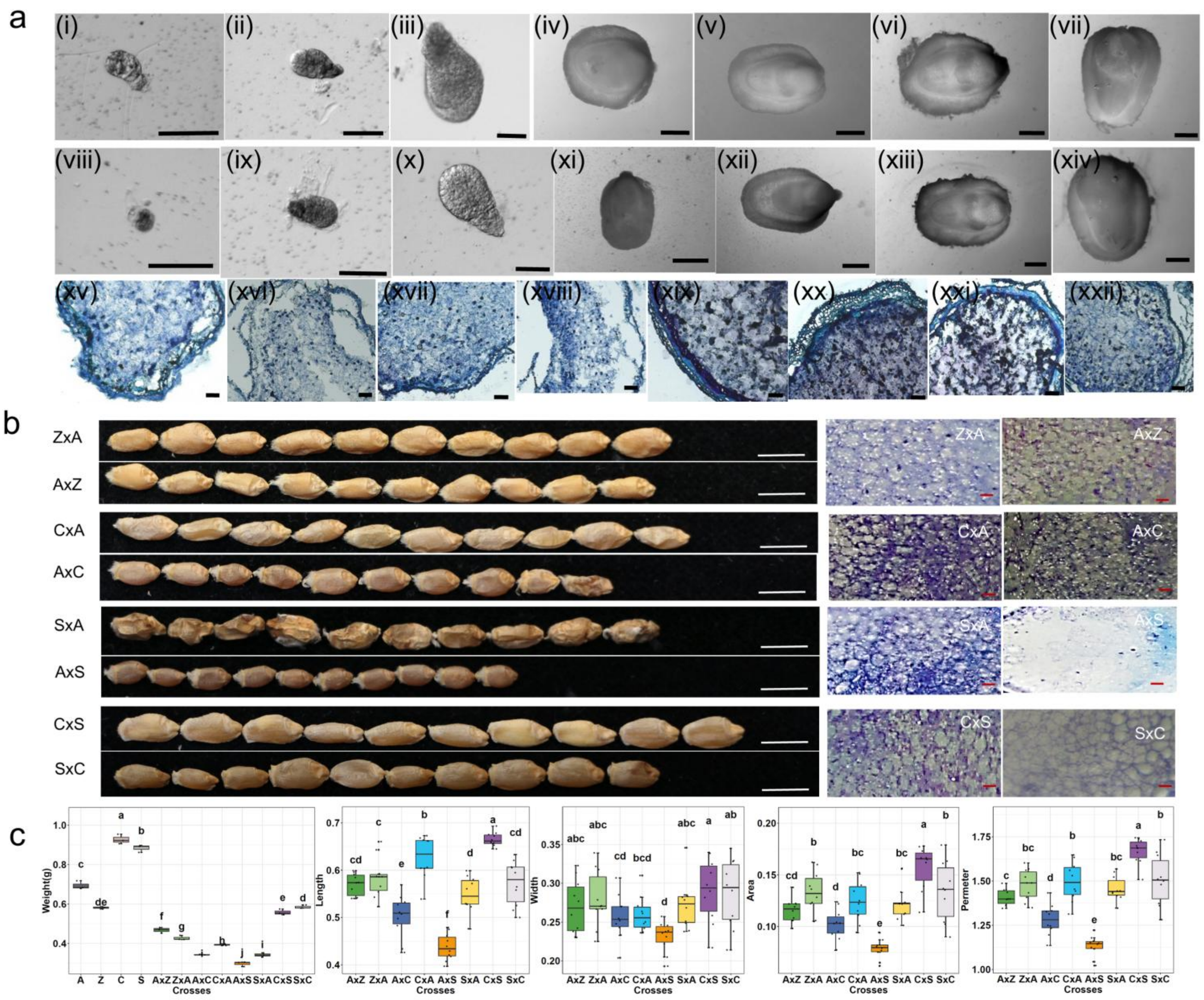
a
(i)
(ii)
(iii)
(iv)
(v)
(vi)
(vii)
(viii)
(ix)
(x)
(xi)
(xii)
(xiii)
(xiv)
(xv)
(xvi)
(xvii)
(xviii)
(xix)
(xx)
(xxi)
(xxii)
b
ZxA
AxZ
CxA
AxC
SxA
AxS
CxS
SxC
c
Weight(g)
Length
Width
Area
Permeter
Crosses

Supplementary Figure 1. Grain development of reciprocal crosses between tetraploid and hexaploid wheats.

(a) Embryo and endosperm development in reciprocal crosses of hexaploid and tetraploid wheats. (i–vii) development embryo of AC Barrie x Strong Field and (viii-xiv) Strong Field x AC Barrie, representative micrographs depict the developmental progression of the wheat embryo from two cell zygote to maturity. (i) and (viii) two cell zygote; (ii) and (ix) pre-embryo; (iii) and (x) transition embryo; (iv) and (xi) leaf early embryo; (v) and (xii) leaf middle embryo; (vi) and (xiii) leaf late embryo; (vii) and (xiv) mature embryo. (xv-xxii) representative Toluidine blue-stained light micrographs depict the developmental progression of the transition stage endosperm (xv) AC Barrie, (xvi) Strong Field, (xvii) AC Barrie x Strong field, (xviii) Strong Field x AC Barrie and leaf late stage endosperm (xix) AC Barrie, (xx) Strong Field, (xxi) AC Barrie x Strong field, (xxii) Strong Field x AC Barrie. Bars: (i, ii, iii, viii, ix, x, xvii-xxii) 100 µm; (iv-vii, xi-xiv) 500 µm.

(b) Phenotyping analysis of mature seeds in reciprocal crosses of hexaploid and tetraploid wheats. Left panel: mature whole seeds of hexaploid and tetraploid. Right panel: representative Toluidine blue-stained light micrographs depict endosperm in the mature seeds. Bars: left panel, 0.5 cm; right panel, 100 um.

(c) Statistical analysis of the grain weight (n = 3), length (n = 10), width (n = 10), area (n = 10) and perimeter (n = 10) in the mature seeds of reciprocal crosses of hexaploid and tetraploid wheats. A, AC Barrie; Z, Chinese Spring; C, Commander; A, Strong Field; AxZ, AC Barrie x Chinese Spring; ZxA, Chinese Spring x AC Barrie; AxC, AC Barrie x Commander; CxA, Commander x AC Barrie; AxS, AC Barrie x Strong Field; SxA, Strong Field x AC Barrie; CxS, Commander x Strong Field; SxC, Strong Field x Commander. The error bar represents the standard deviation for each sample, the bottom and top of the box are the 25th and 75th percentiles, the line inside the box is the 50th percentile (median). Statistical analysis was conducted by one-way ANOVA. Samples without the same letter have significant difference in expression levels (p value <0.05).

a

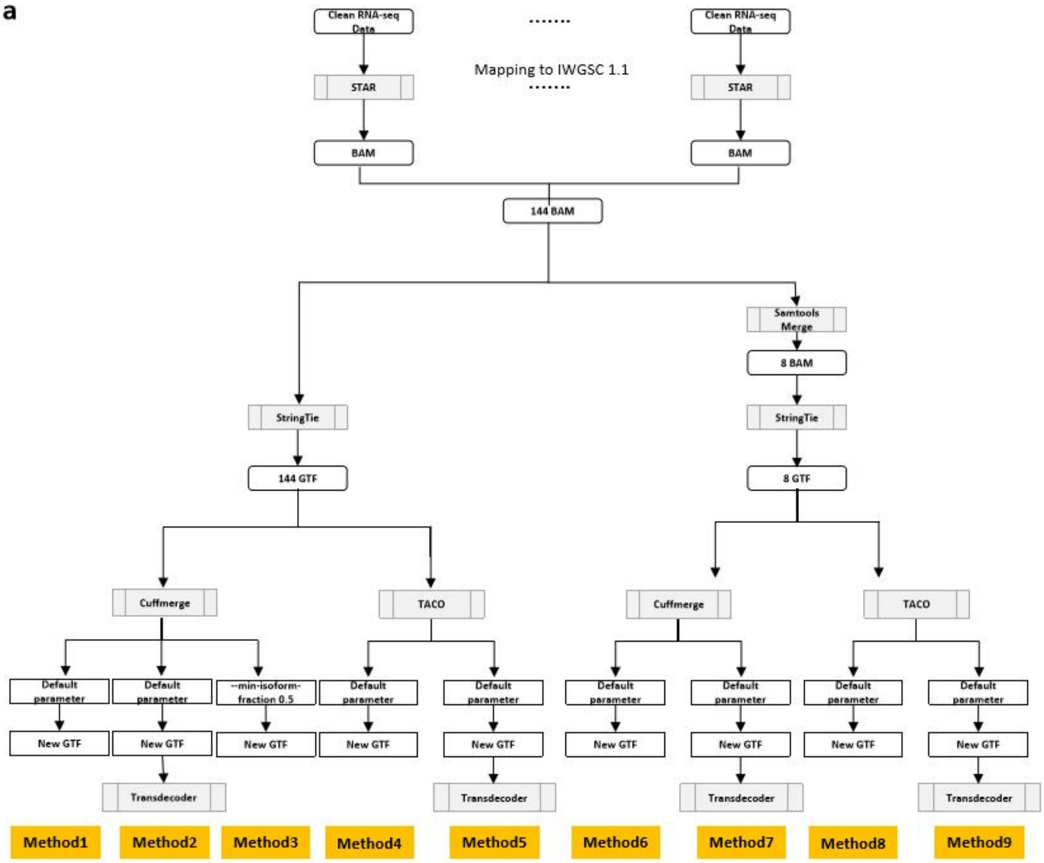

b

	Standard strand			unstandard strand	Total gene number	Number of Genes overlap with RefSeq 1.1
	Total	positive strand	negative strand			
Method1	169,664	85,251	84,413	64,854	234,518	106,743
Method2	126,508	63,440	63,068	-	126,508	99,327
Method3	169,850	85,331	84,519	64,802	234,652	106,879
Method4	143,975	72,293	71,682	-	143,975	74,138
Method5	44,607	22,493	22,114	-	44,607	31,850
Method6	185,048	92,979	92,069	156,001	341,049	106,536
Method7	146,418	73,376	73,042	-	146,418	99,257
Method8	80,337	40,309	40,028	-	80,337	58,756
Method9	63,889	32,052	31,837	-	63,889	54,338

c

	Method1		Method2		Method3		Method4		Method5		Method6		Method7		Method8		Method9	
	Sensitivity %	Precision %	Sensitivity %	Precision %	Sensitivity %	Precision %	Sensitivity %	Precision %	Sensitivity %	Precision %	Sensitivity %	Precision %	Sensitivity %	Precision %	Sensitivity %	Precision %	Sensitivity %	Precision %
Base level	100	57.5	98	74.6	100	58	48.7	54.7	30.4	59.9	100	40.9	98.1	61.9	55.9	61.6	54.5	70.8
Exon level	100	47.6	97.7	64.2	100	49	41.6	33.3	30.3	61.2	100	44.5	97.7	65	54.8	60.3	53.9	69.5
Intron level	100	52.8	99	65.6	100	54.9	51.5	57.7	36.5	74.8	100	56.8	99	71	64.7	74.7	63.9	83
Intron chain level	100	22.9	97	28.6	100	25.2	17.1	6.9	14.1	25.2	100	26.7	97	34.7	30.2	25.8	29.2	29.1
Transcript level	100	23.9	93.9	31.8	100	25.8	21.3	8.2	13.4	26.8	100	22.7	94	36	28.9	27.7	27.4	31.3
Locus level	100	45.5	92.6	78.3	100	45.5	25.7	19.3	16.3	39.4	100	31.2	92.7	67.6	34.6	46.5	32.8	55.4

Supplementary Figure 2 Construction of a consensus transcriptome from multiple samples with different ploidy levels. (**a**) Assembly methods and mapping details. StringTie was used for generating reference-guide GTF file and new annotation predicted by Cuffmerge or TACO. Transdecoder was used for new gene annotation further filter by finding coding

regions within transcripts. Two different input BAM files dealing (merged by sample or not) generated and nine different procedures were tested. Method1: StringTie+Cuffmerge[default para]; Method2: StringTie+Cuffmerge[default para]+TransDecoder; Method3: StringTie+Cuffmerge[para : --min-isoform-fraction 0.5]; Method4: StringTie+TACO; Method5: StringTie+TACO+TransDecoder; Method6: Merged BAM+StringTie+Cuffmerge; Method7: Merged BAM+StringTie+Cuffmerge+TransDecoder; Method8: Merged BAM+StringTie+TACO; Method9: Merged BAM+StringTie+TACO+TransDecoder.

(b) Number of genes identified in each of the nine method. The genes overlapped with the IWGSCv1.1 annotated genes and newly identified genes in different methods were counted.

(c) Mapping rate (%) and precision rate (%) of different methods.

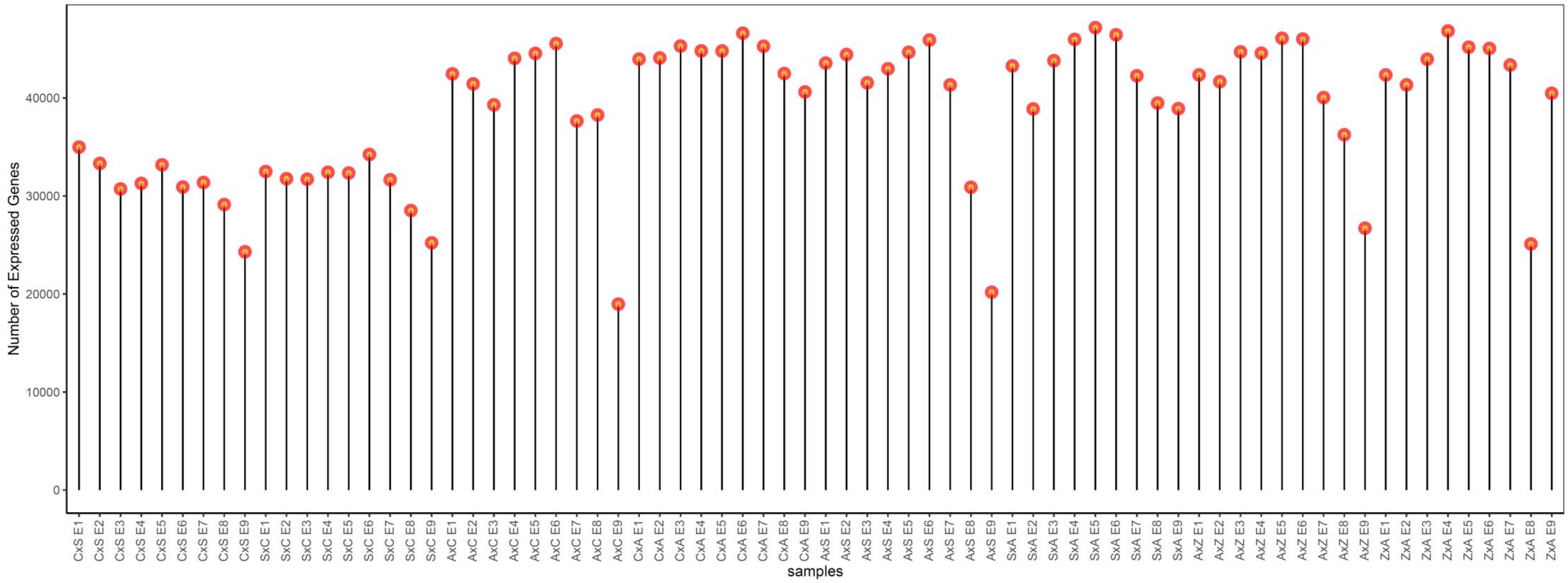

Supplementary Figure 3 Number of expressed genes detected at each stage (TPM>=1) in each sample. Genes with more than one TPM in at least one of all samples were considered expressed. TPM, transcripts per million.

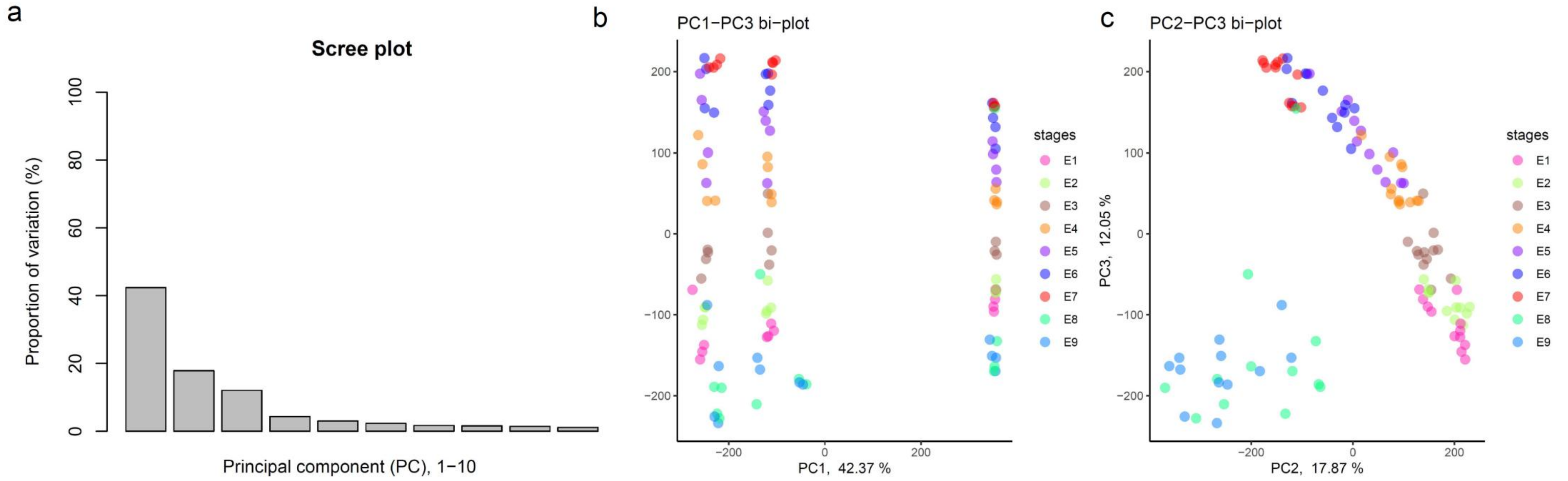

Supplementary Figure 4 Principal component analysis (PCA) using TPM values of all genes from 144 samples. TPM values were mean of two replicates for each sample. **a**, Proportion of variation (%) of top 10 PCA components. 2D PCA plots were generated for PC1-PC3 (b) and PC2-PC3 (c). Proportion of variance for each principal component is indicated in x axis titles. Seven stages of embryo development (from zygote to mature embryo, E1 to E7), and two stages of endosperm (early and late stage, E8 and E9) are labeled with different colors.

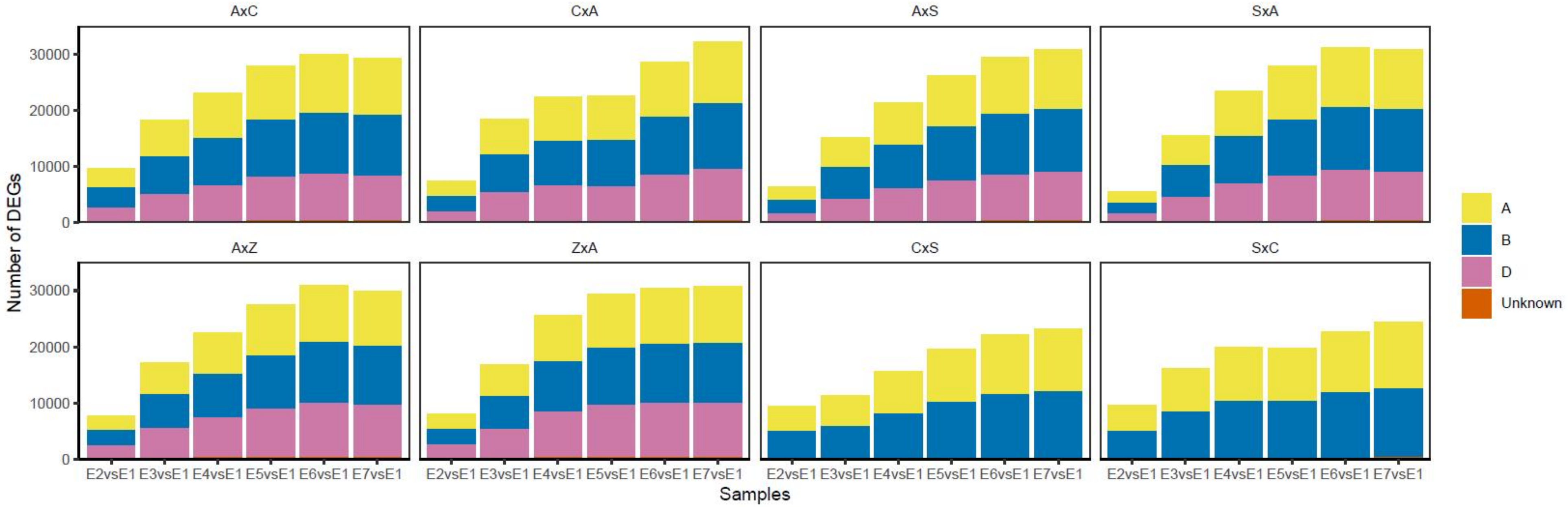

Supplementary Figure 5 Number of differentially expressed genes (DEGs) during embryo development. DEGs were identified through comparisons between each stage against the two-cell stage (E1) for each sample. Genes with an adjusted p-value < 0.01 and log2 fold change >= 1 or =< -1 were considered DEGs. Number of DEGs from each comparison in the F1 hybrids of the reciprocal crosses AxC, CxA, AxS, SxA, AxZ, ZxA, CxS and SxC are shown. CxS, Commander x Strong Field; SxC, Strong Field x Commander; CxA, Commander x AC Barrie; AxC, AC Barrie x Commander; SxA, Strong Field x AC Barrie; AxS, AC Barrie x Strong Field; ZxA, Chinese Spring x AC Barrie; AxZ, AC Barrie x Chinese Spring. X axis represents the comparisons between each stage against E1 stage. Y axis represents the number of DEGs.

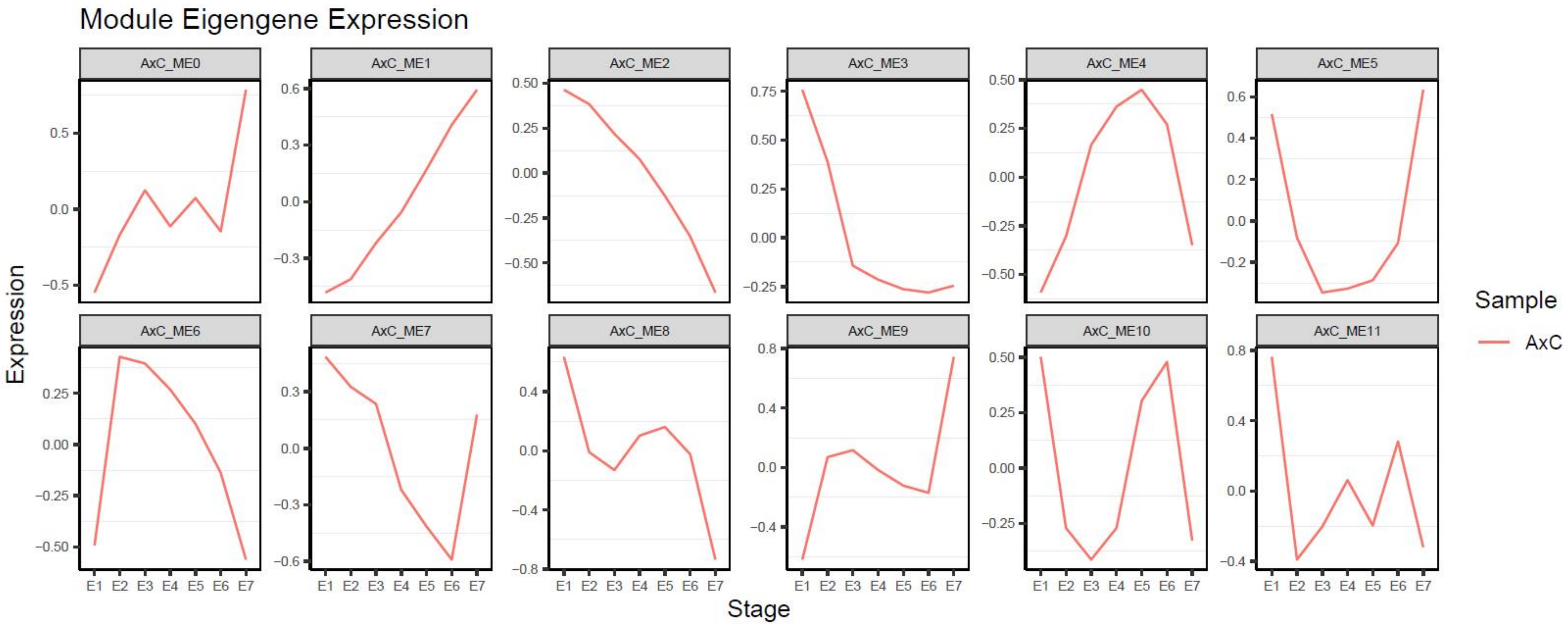

Supplementary Figure 6 Module eigengene values of clusters identified in AC Barrie x Commander (AxC). Differentially expressed genes (DEGs) were clustered with weighted correlation network analysis (WGCNA) to identify clusters using a dynamic hierarchical clustering approach. The module eigengenes (MEs) which represent the first principal component of each WGCNA module were plotted to indicate the expression pattern of each cluster. X axis represents ME values while y axis indicate embryo stages.

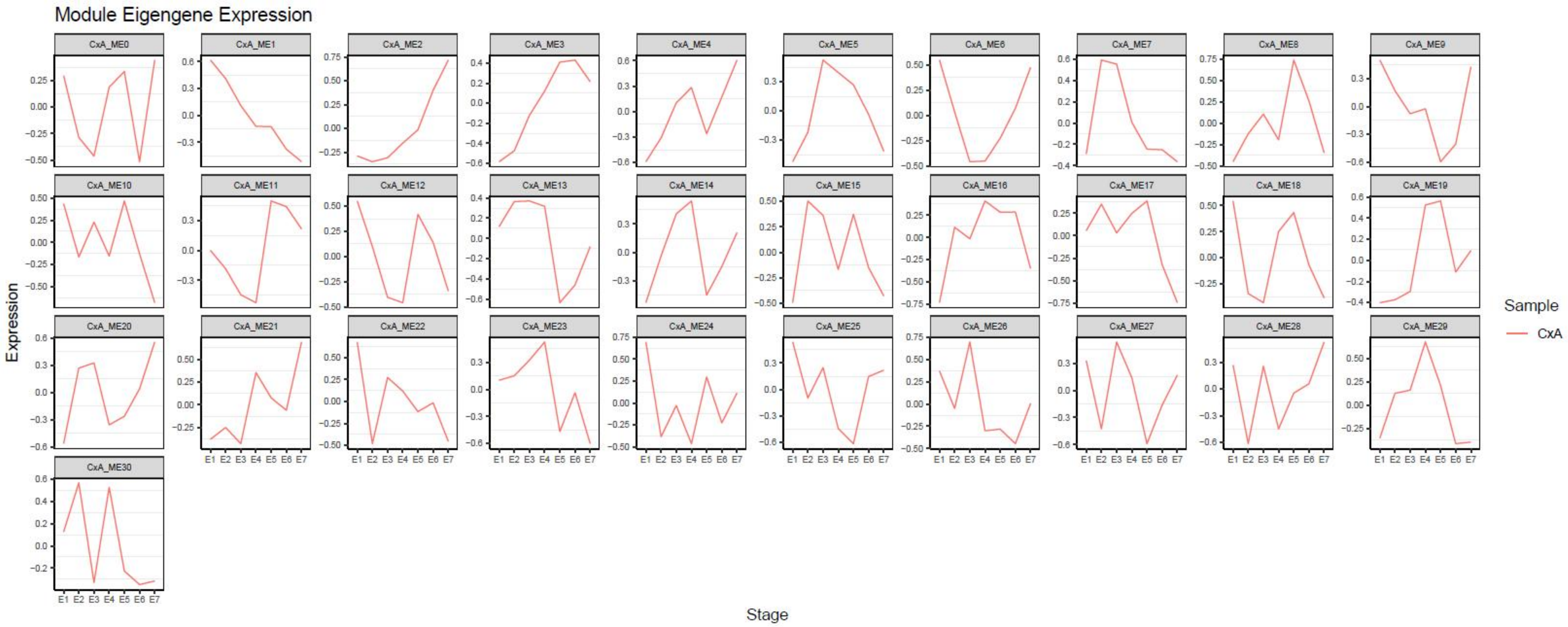

Supplementary Figure 7 Module eigengene values of clusters identified in Commander x AC Barrie (CxA). Differentially expressed genes (DEGs) were clustered with weighted correlation network analysis (WGCNA) to identify clusters using a dynamic hierarchical clustering approach. The module eigengenes (MEs) which represent the first principal component of each WGCNA module were plotted to indicate the expression pattern of each cluster. X axis represents ME values while y axis indicate embryo stages.

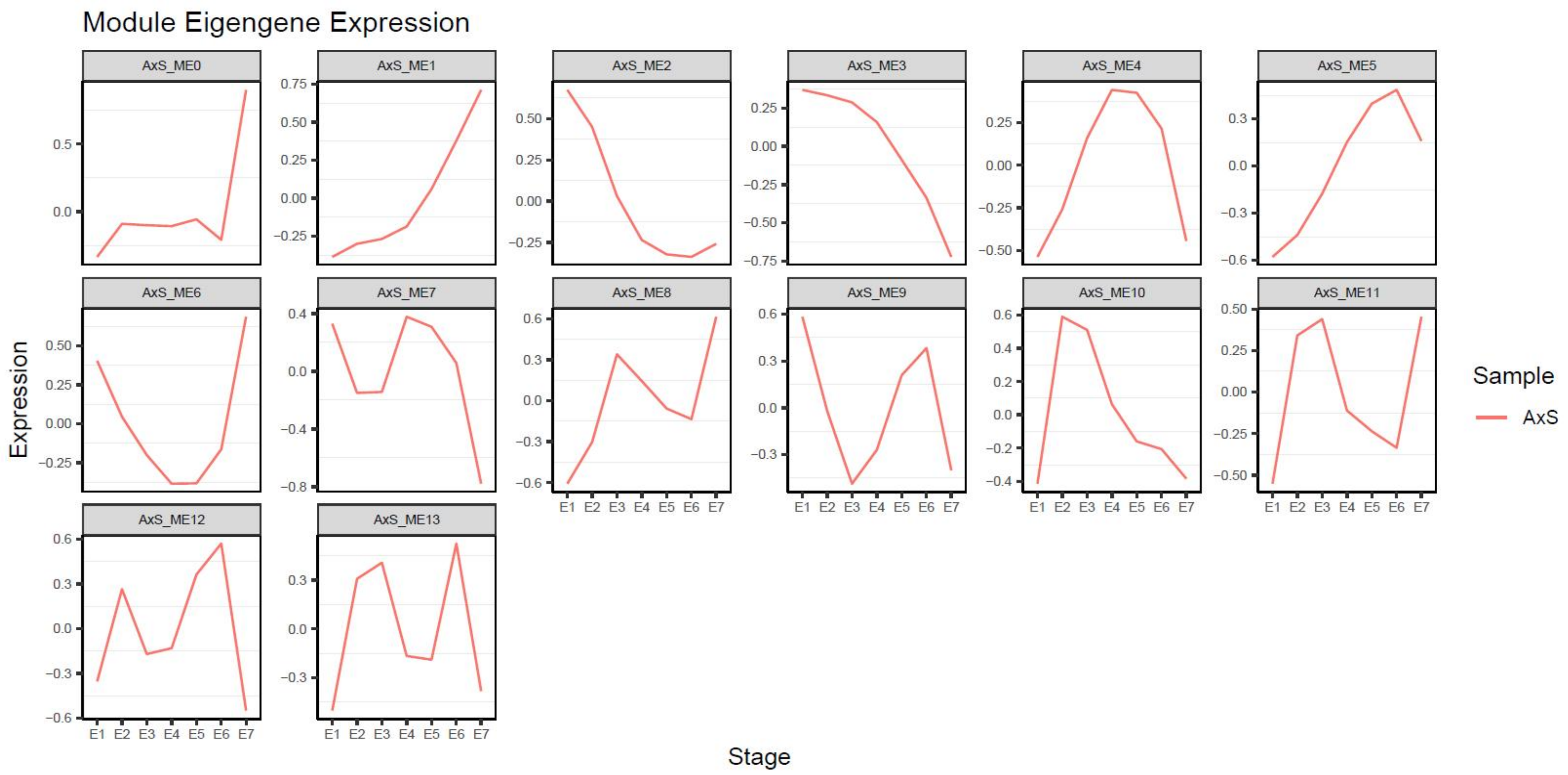

Supplementary Figure 8 Module eigengene values of clusters identified in AC Barrie x Strong Field (AxS). Differentially expressed genes (DEGs) were clustered with weighted correlation network analysis (WGCNA) to identify clusters using a dynamic hierarchical clustering approach. The module eigengenes (MEs) which represent the first principal component of each WGCNA module were plotted to indicate the expression pattern of each cluster. X axis represents ME values while y axis indicate embryo stages.

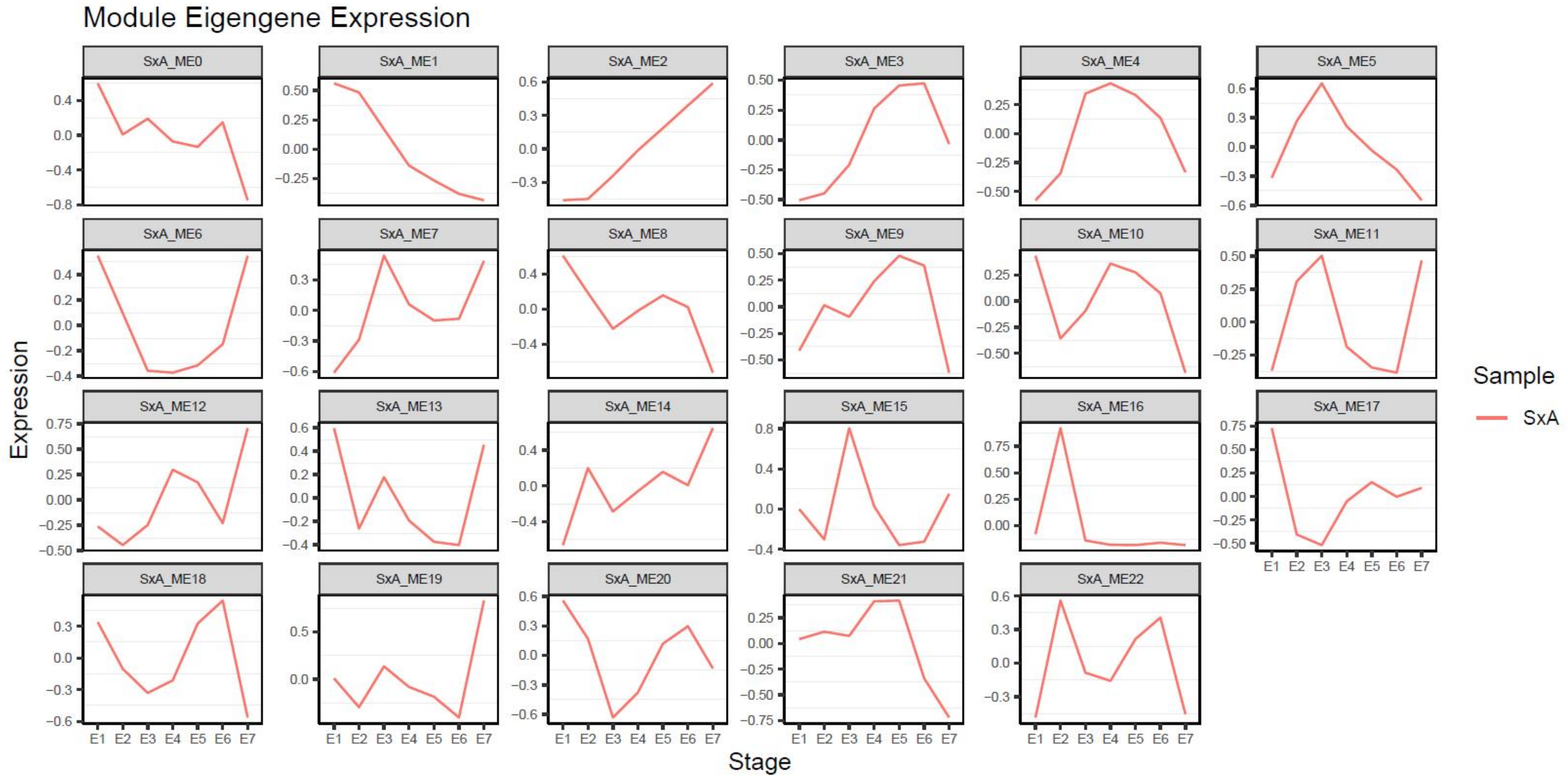

Supplementary Figure 9 Module eigengene values of clusters identified in Strong Field x AC Barrie (SxA). Differentially expressed genes (DEGs) were clustered with weighted correlation network analysis (WGCNA) to identify clusters using a dynamic hierarchical clustering approach. The module eigengenes (MEs) which represent the first principal component of each WGCNA module were plotted to indicate the expression pattern of each cluster. X axis represents ME values while y axis indicate embryo stages.

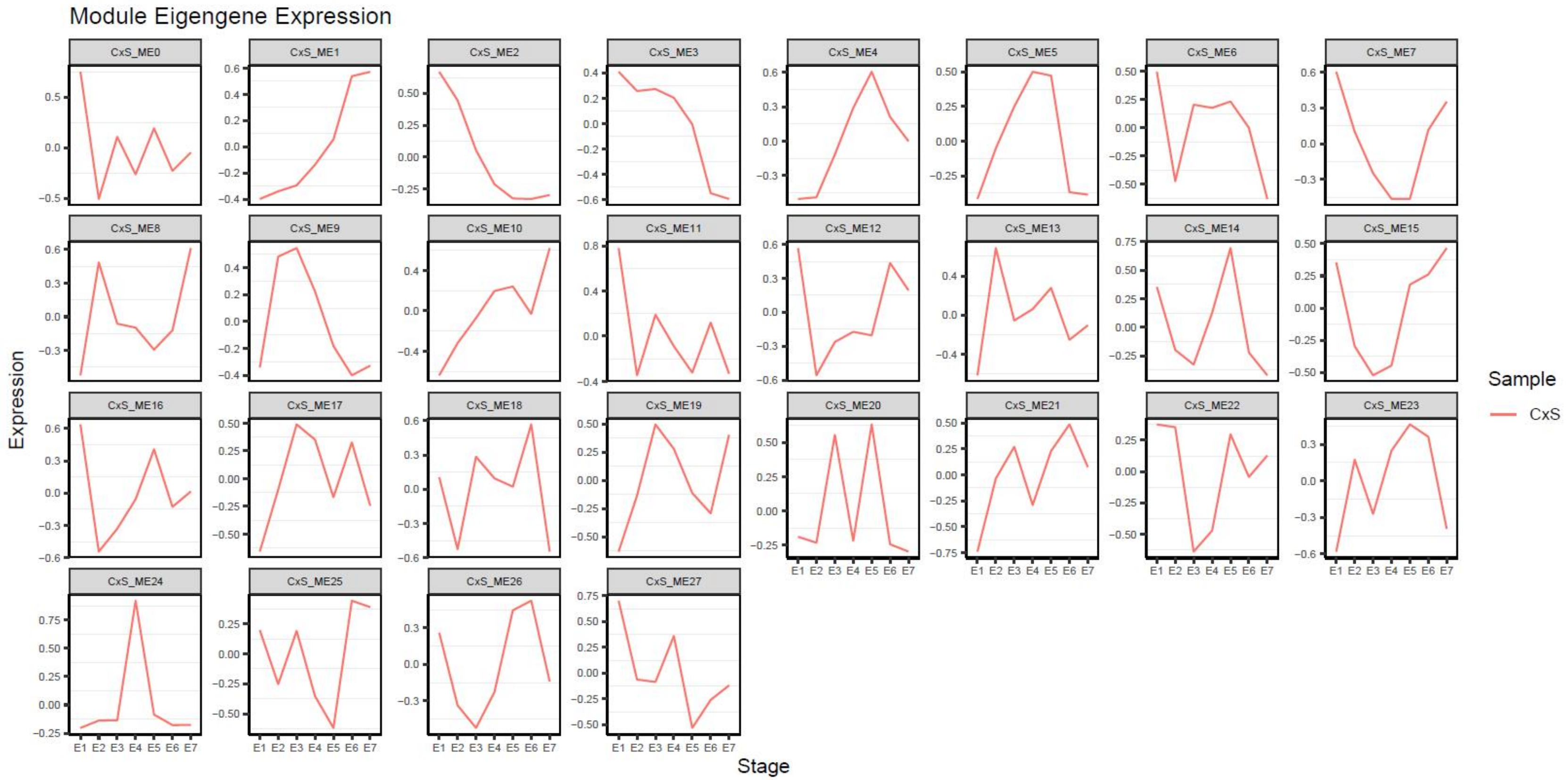

Supplementary Figure 10 Module eigengene values of clusters identified in Commander x Strong Field (CxS). Differentially expressed genes (DEGs) were clustered with weighted correlation network analysis (WGCNA) to identify clusters using a dynamic hierarchical clustering approach. The module eigengenes (MEs) which represent the first principal component of each WGCNA module were plotted to indicate the expression pattern of each cluster. X axis represents ME values while y axis indicate embryo stages.

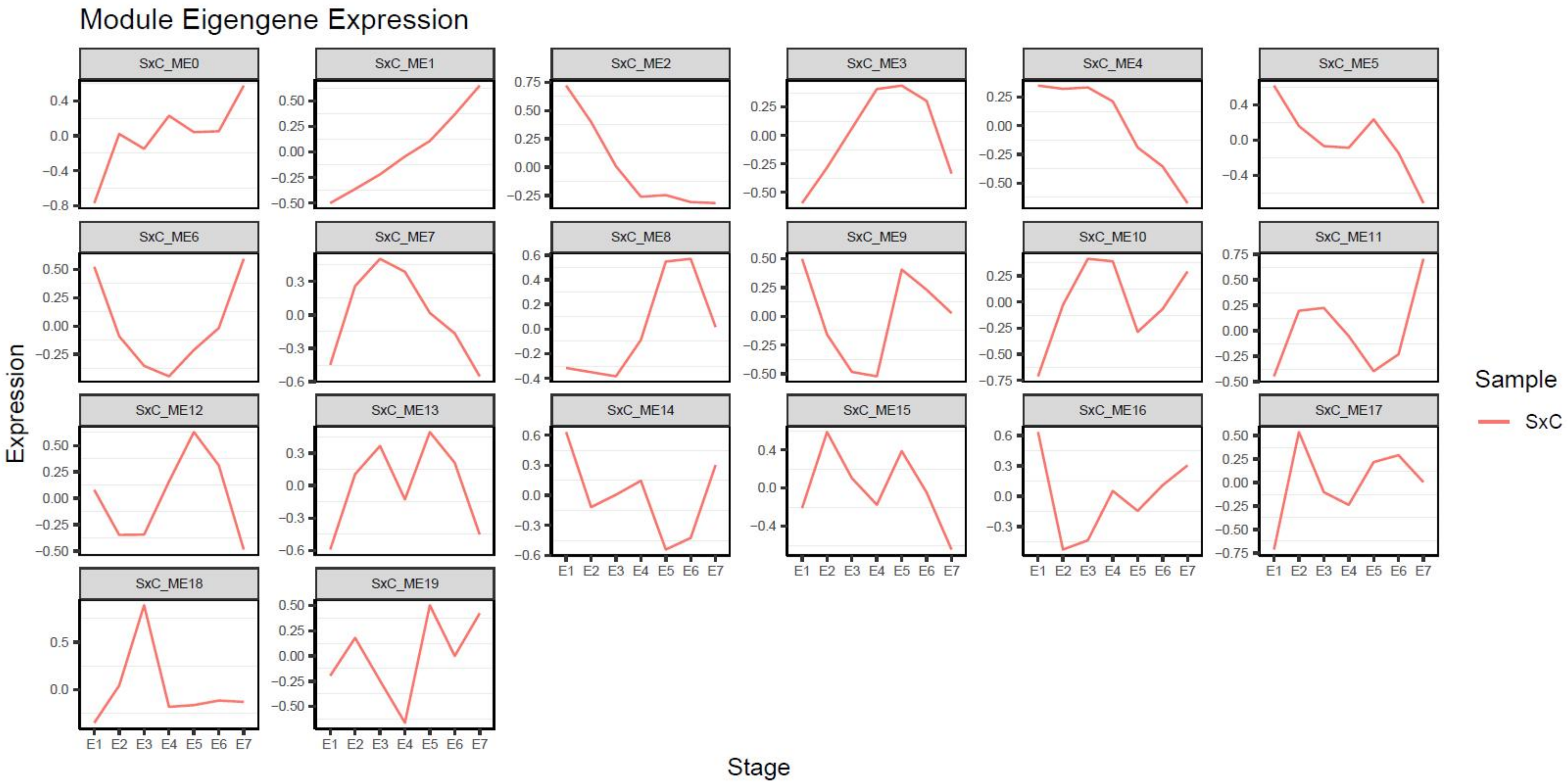

Supplementary Figure 11 Module eigengene values of clusters identified in Strong Field x Commander (SxC). Differentially expressed genes (DEGs) were clustered with weighted correlation network analysis (WGCNA) to identify clusters using a dynamic hierarchical clustering approach. The module eigengenes (MEs) which represent the first principal component of each WGCNA module were plotted to indicate the expression pattern of each cluster. X axis represents ME values while y axis indicate embryo stages.

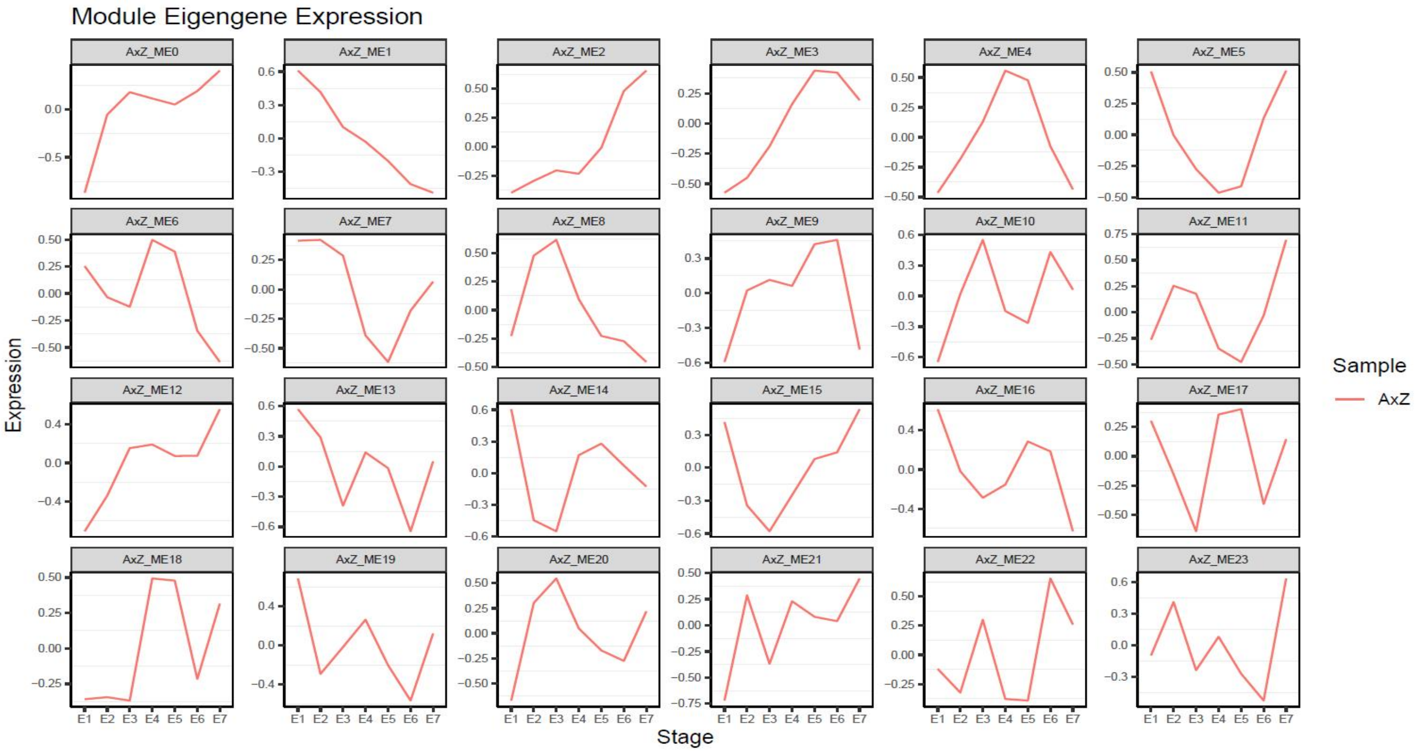

Supplementary Figure 12 Module eigengene values of clusters identified in AC Barrie x Chinese Spring (AxZ). Differentially expressed genes (DEGs) were clustered with weighted correlation network analysis (WGCNA) to identify clusters using a dynamic hierarchical clustering approach. The module eigengenes (MEs) which represent the first principal component of each WGCNA module were plotted to indicate the expression pattern of each cluster. X axis represents ME values while y axis indicate embryo stages.

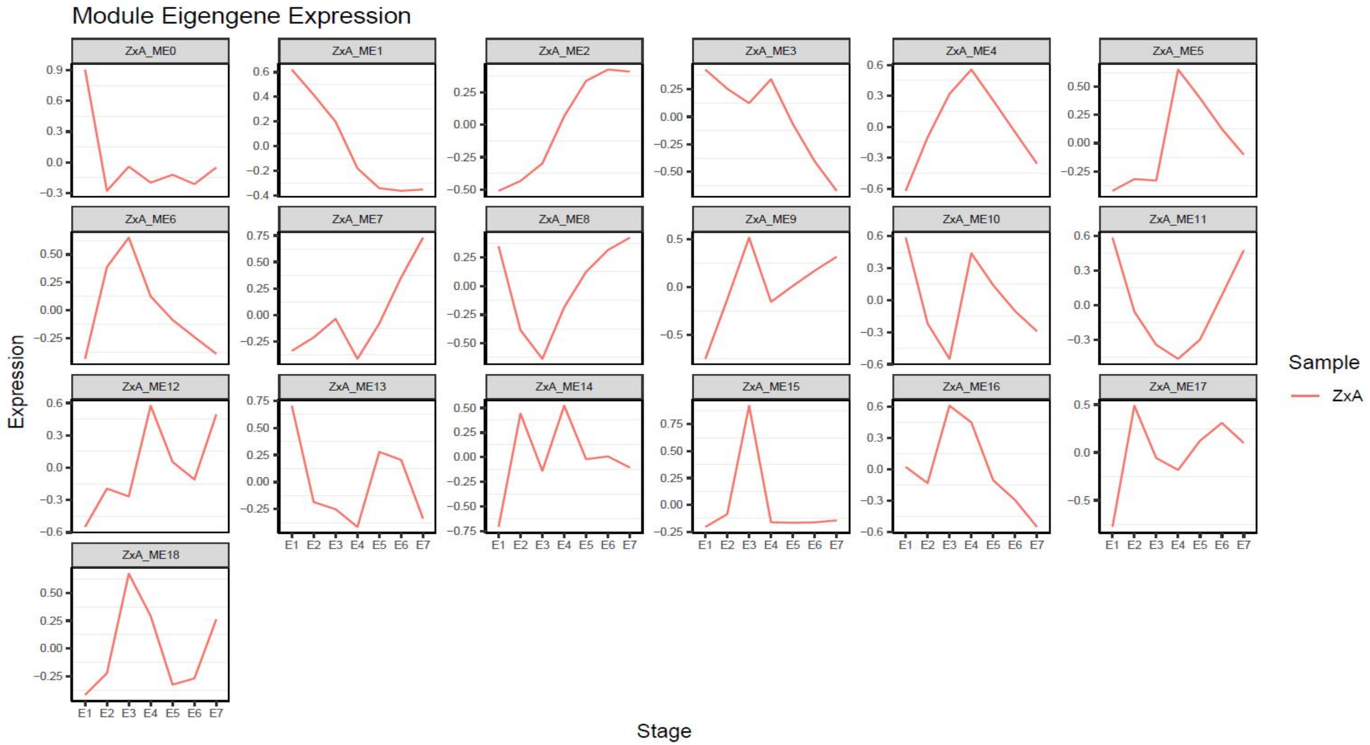

Supplementary Figure 13 Module eigengene values of clusters identified in Chinese Spring x AC Barrie (ZxA). Differentially expressed genes (DEGs) were clustered with weighted correlation network analysis (WGCNA) to identify clusters using a dynamic hierarchical clustering approach. The module eigengenes (MEs) which represent the first principal component of each WGCNA module were plotted to indicate the expression pattern of each cluster. X axis represents ME values while y axis indicate embryo stages.

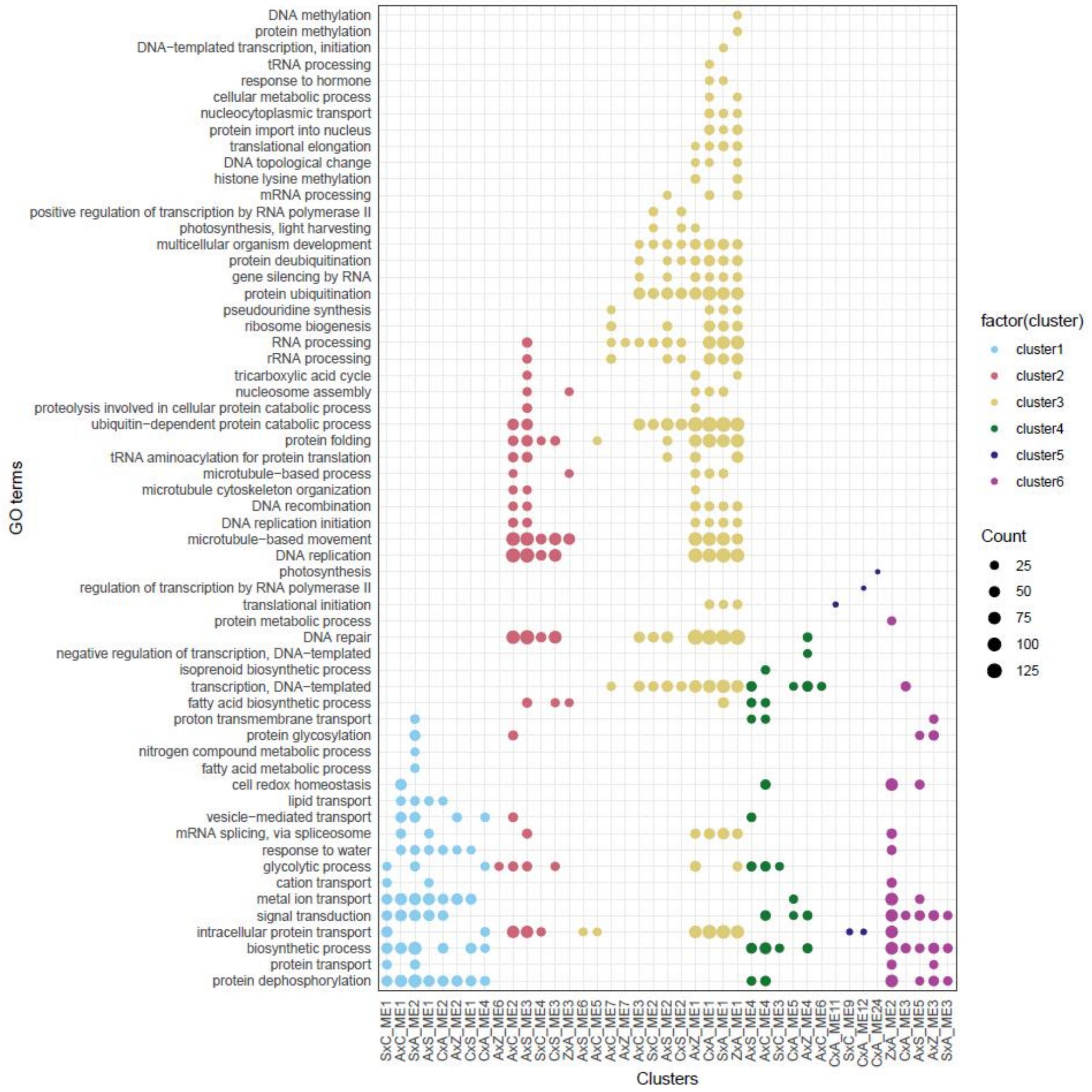

Supplementary Figure 14 Go enrichment analysis for clusters identified in different reciprocal crosses. Differentially expressed genes (DEGs) in each sample was clustered separately and GO enrichment analysis was performed for each clustered in each sample. A total of 11, 30, 13, 22, 27, 19, 23 and 18 clusters were identified AxC, CxA, AxS, SxA, CxS, SxC, AxZ and ZxA, respectively. The module eigengene (ME)values which represent the first principal component for each cluster were reclustered into six clusters and highly enriched GO terms from each cluster were plotted (FDR < 0.05). MEs belongs to different clusters were colored. Dot size represent number of genes in each GO term (only those with more than 20 genes are shown in clusters). X axis represents the clusters as indicated by sample name and the number of ME. Y axis are GO terms.

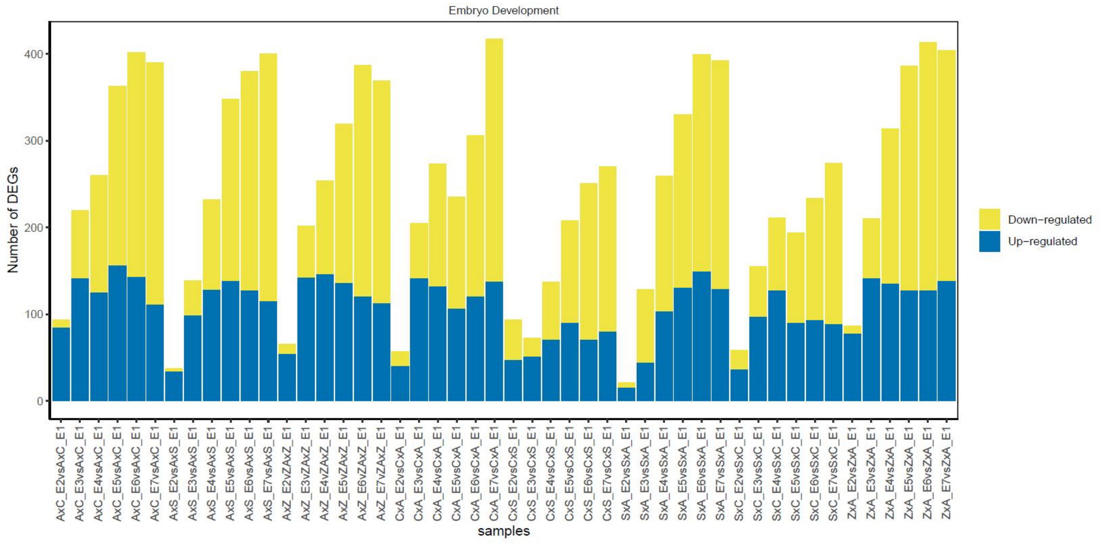

Supplementary Figure 15 Number of DEGs in embryo development essential pathways during embryo development. DEGs were identified through comparisons between each stage against the two-cell stage (E1) for each sample. Genes with an adjusted p-value < 0.01 and log2 fold change >= 1 or =< -1 were considered DEGs. Number of DEGs from each comparison in the F1 hybrids of the reciprocal crosses AxC, CxA, AxS, SxA, AxZ, ZxA, CxS and SxC are shown. CxS, Commander x Strong Field; SxC, Strong Field x Commander; CxA, Commander x AC Barrie; AxC, AC Barrie x Commander; SxA, Strong Field x AC Barrie; AxS, AC Barrie x Strong Field; ZxA, Chinese Spring x AC Barrie; AxZ, AC Barrie x Chinese Spring. X axis represents the comparisons between each stage against E1 stage. Y axis shows the number of DEGs involved in embryo development essential pathways summarized from Xiang et al., 2019 (Supplementary Figure 19 and Data S7).

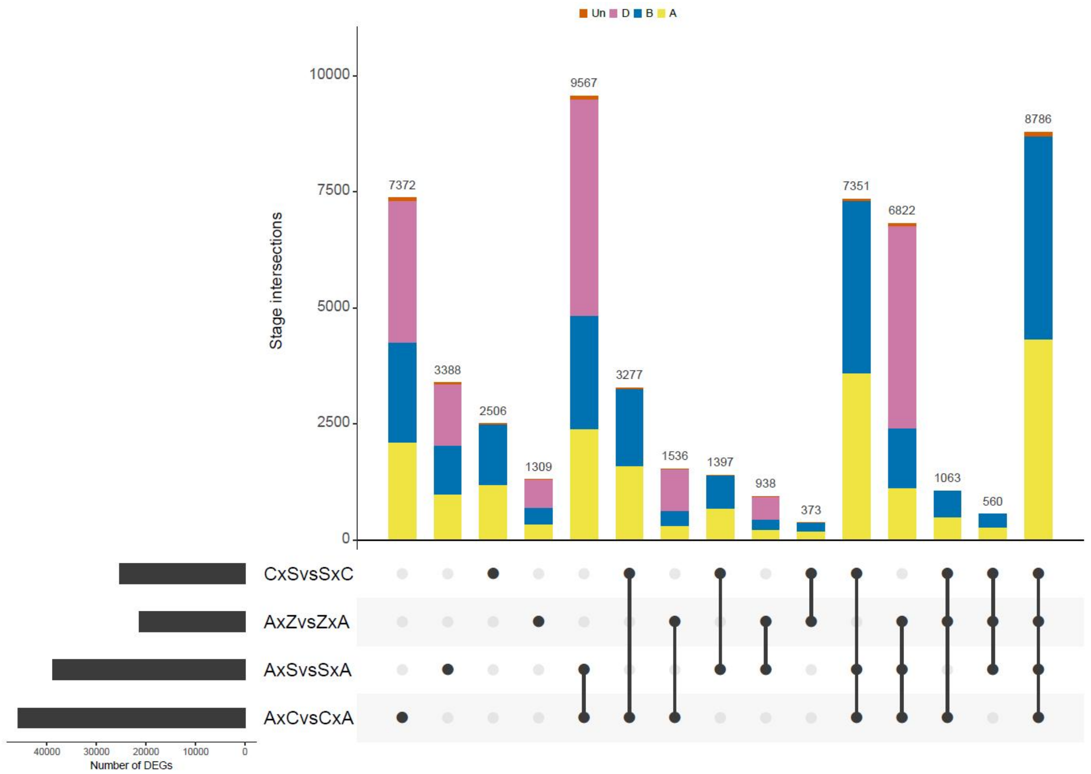

Supplementary Figure 16 Number of DEGs shared between crosses. The numbers of DEGs shared between four pairs of reciprocal crosses (AxC vs CxA, AxS vs SxA, CxS vs SxC and AxZ vs ZxA) are shown by Upset plot. Genes from A, B, D and unknown (Un) genome are plotted as different colors.

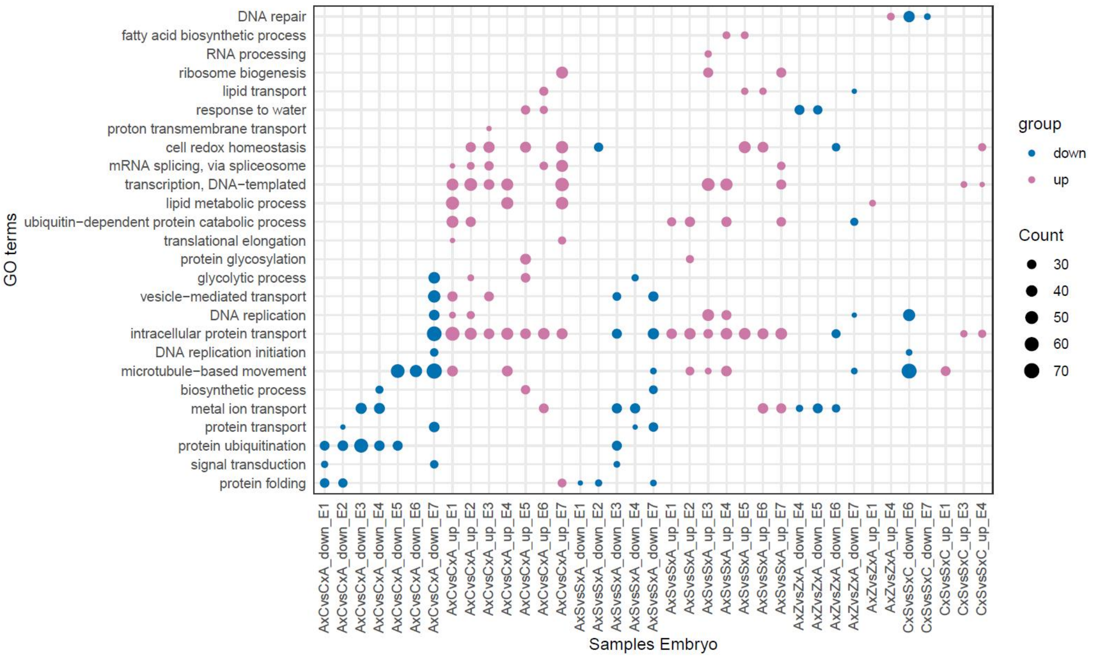

Supplementary Figure 17 Go enrichment analysis for the up-regulated and down-regulated genes between reciprocal pairs in embryos. Up-regulated DEGs (cherry color dots) represent genes that are highly expressed in AxC against CxA, AxS against SxA, AxZ against ZxA and CxS against SxC (log2FC > 1 or <-1 and FDR<0.01). Down-regulated DEGs (blue color dots) represent genes that are expressed at lower levels in AxC against CxA, AxS against SxA, AxZ against ZxA and CxS against SxC (log2FC > 1 or <-1 and FDR<0.01). Dot size represent number of genes in each GO term (only those with more than 20 counts are shown).

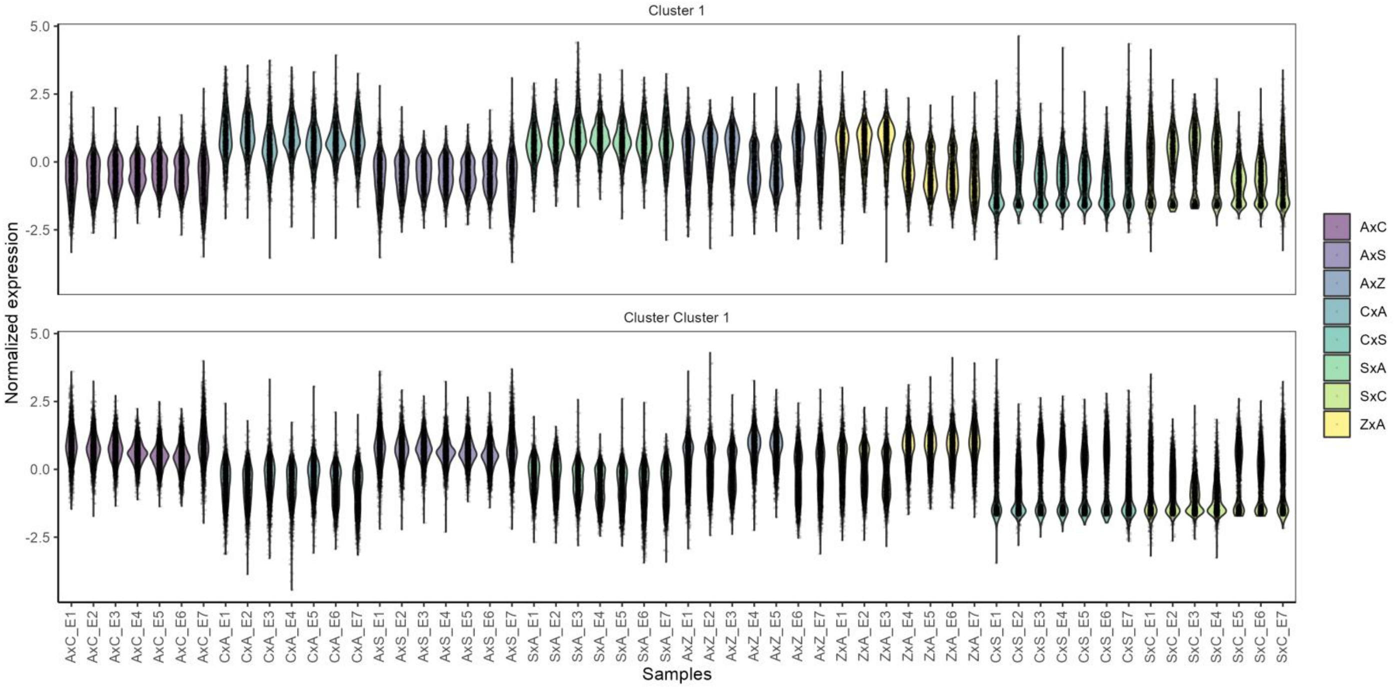

Supplementary Figure 18 Normalized expression of differentially expressed genes between reciprocal crosses across all stages. Differentially expressed genes were identified through ANOVA test (p value < 0.05) and categorized into two groups. Cluster 1 shows genes with higher expression levels in pentaploids whose female parent is tetraploid compared to those whose female parent is hexploid. Cluster 2 shows genes with higher expression levels in pentaploids whose female parent is hexploid compared to those whose female parent is tetraploid.

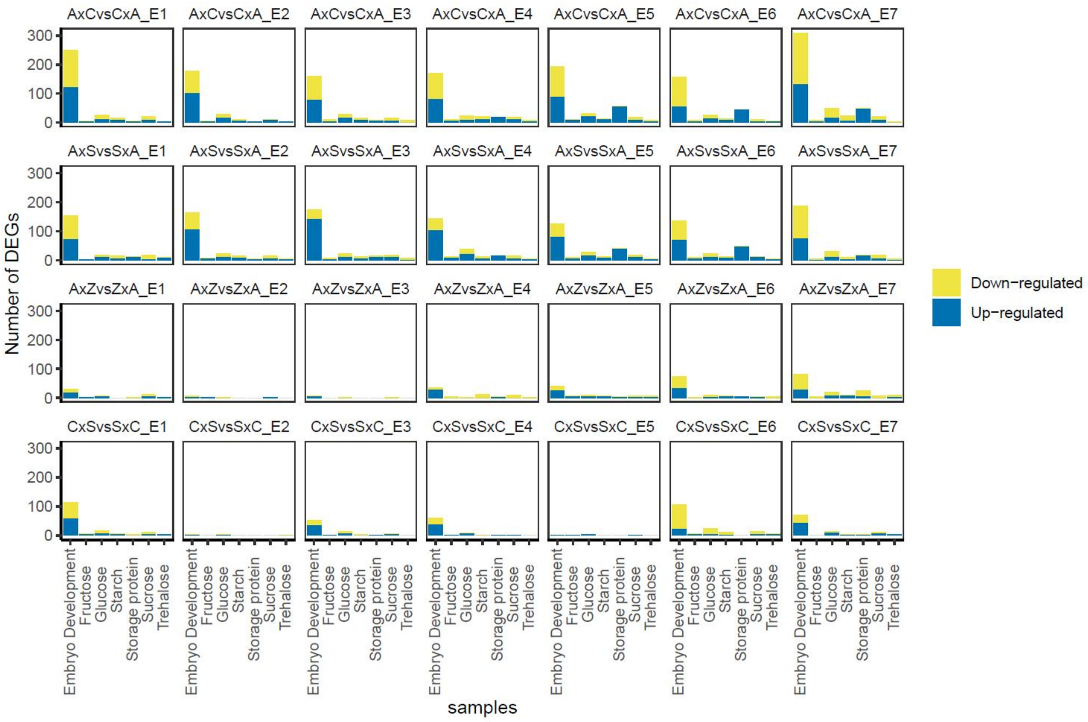

Supplementary Figure 19 Number of DEGs in embryo development essential pathways between reciprocal crosses. DEGs were identified through comparisons between AxC against CxA, AxS against SxA, AxZ against ZxA and CxS against SxC, at each of the seven embryo stages. Genes with an adjusted p-value < 0.01 and log2 fold change >= 1 or =< -1 were considered DEGs. X axis represents the comparisons in each reciprocal cross pair at each stage from E1 to E7. Y axis shows the number of DEGs involved in embryo development essential pathways summarized from Xiang et al., 2019 (Supplementary Figure 19 and Data S7).

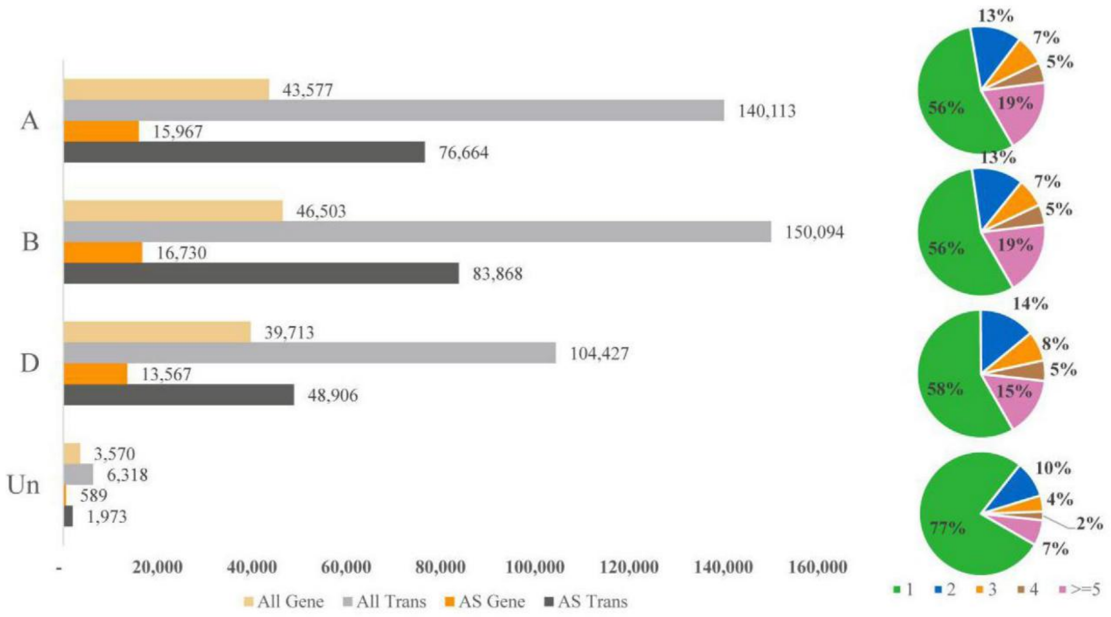

Supplementary Figure 20 Summary of alternative splicing events identified in reciprocal crosses between hexploid and tetraploid wheats. AS events were detected from all samples according to the annotated isoforms in the IWGSC ref1.1 genome using SUPPA2 (Trincado et al., 2018). Column diagrams shows the number of assembled gene (yellow), transcripts (light grey), alternative spliced genes (orange) and AS transcripts (dark grey) on each subgenome. Pie charts show the proportion of total assembled genes with single-transcript or multiple transcripts. Green, blue, yellow, brown and purple color represent genes with one, two, three, four and greater than five transcripts.

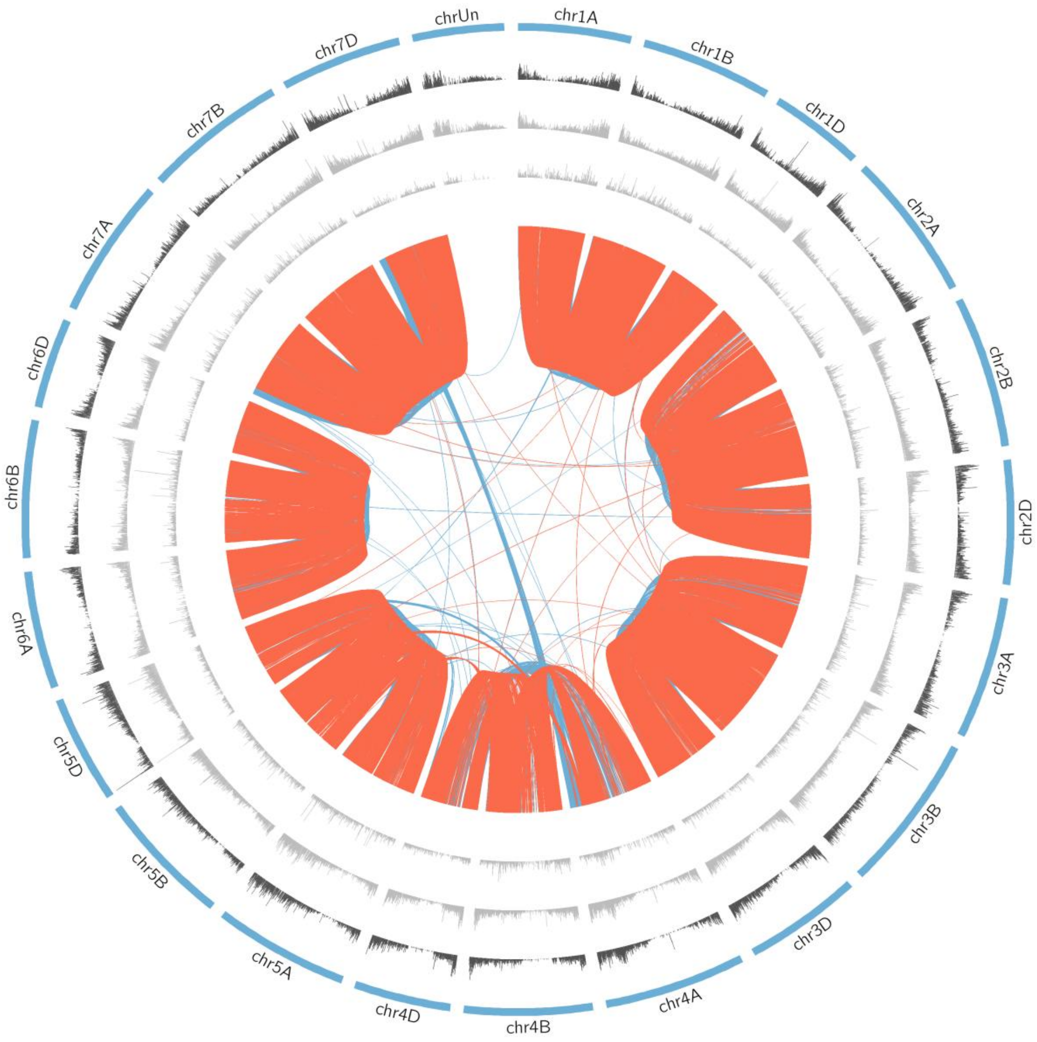

Supplementary Figure 21 Distribution of genes, alternative splicing (AS) events and homeologous triads on wheat chromosomes identified in all samples in this study. Chromosome distribution of genes, AS and homeologous triads identified in this study were plotted. From outside lane to inside lane are gene density in reference genome, genes identified in this study, AS events and homeologous genes. Orange color links represent triads (homeologous genes on A, B and D genomes) while blue color links are duplets (homeologous genes on two of the A, B and D genomes) are shown.

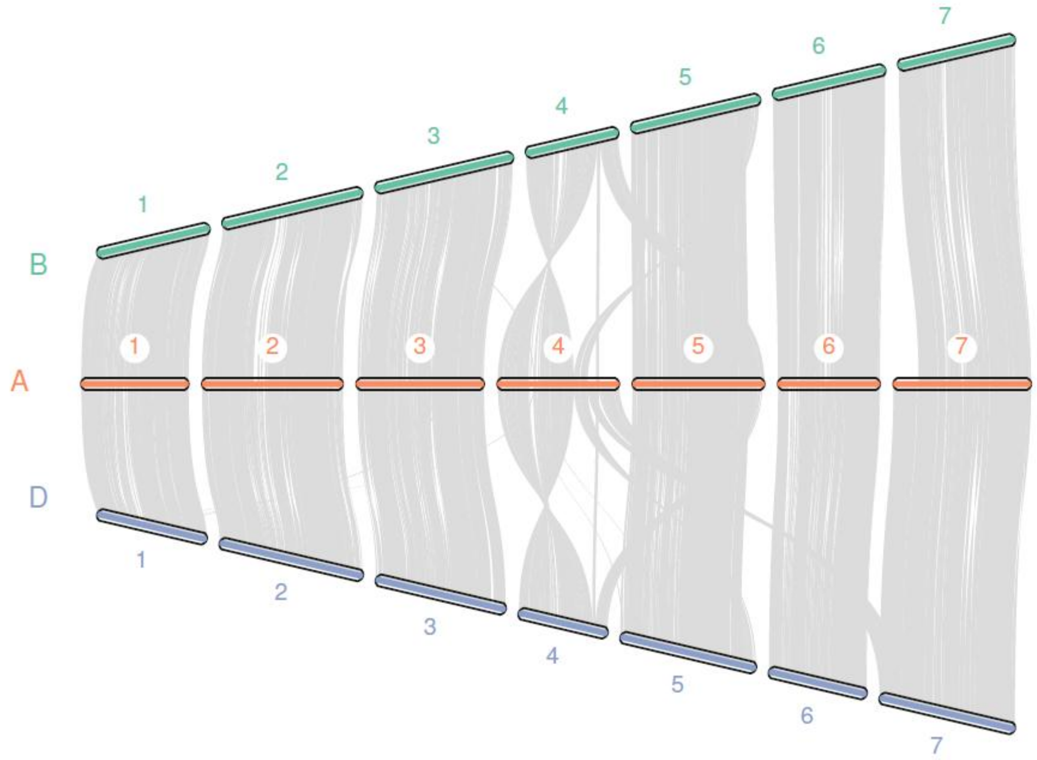

Supplementary Figure 22 Synteny plot of homeologous triads on wheat chromosomes. Homoeologous genes in A, B and D subgenome from all samples in this study were identified using the longest transcripts seq by pairwise synteny search using MCScan with default parameters (Wang et al., 2012; Tang et al., 2008). Homeologous triads and gene order between A (orange color), B (green color) and D (blue color) genomes are shown with synteny plot. Numbers on each bar represent the number of chromosome in each genome.

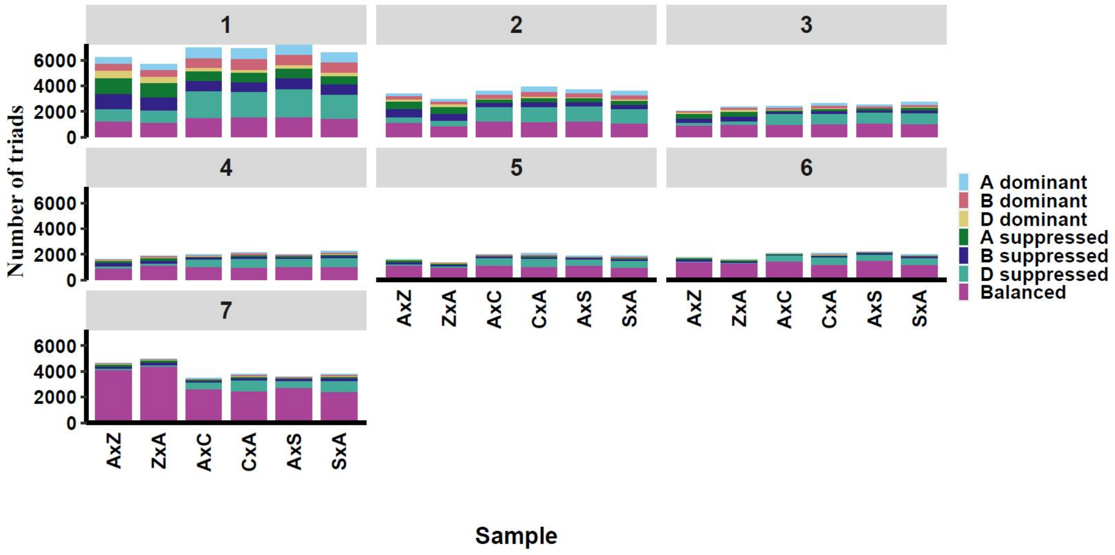

Supplementary Figure 23 Number of triads in each category across embryo stages. Triads were classified based on the percent valuesinto seven groups including balanced, A dominant, B dominant, D dominant, A suppressed, B suppressed and D suppressed, as indicated by different colors. Number of triads and their classification in one, two, three, four, five, six and seven embryo stages were plotted.

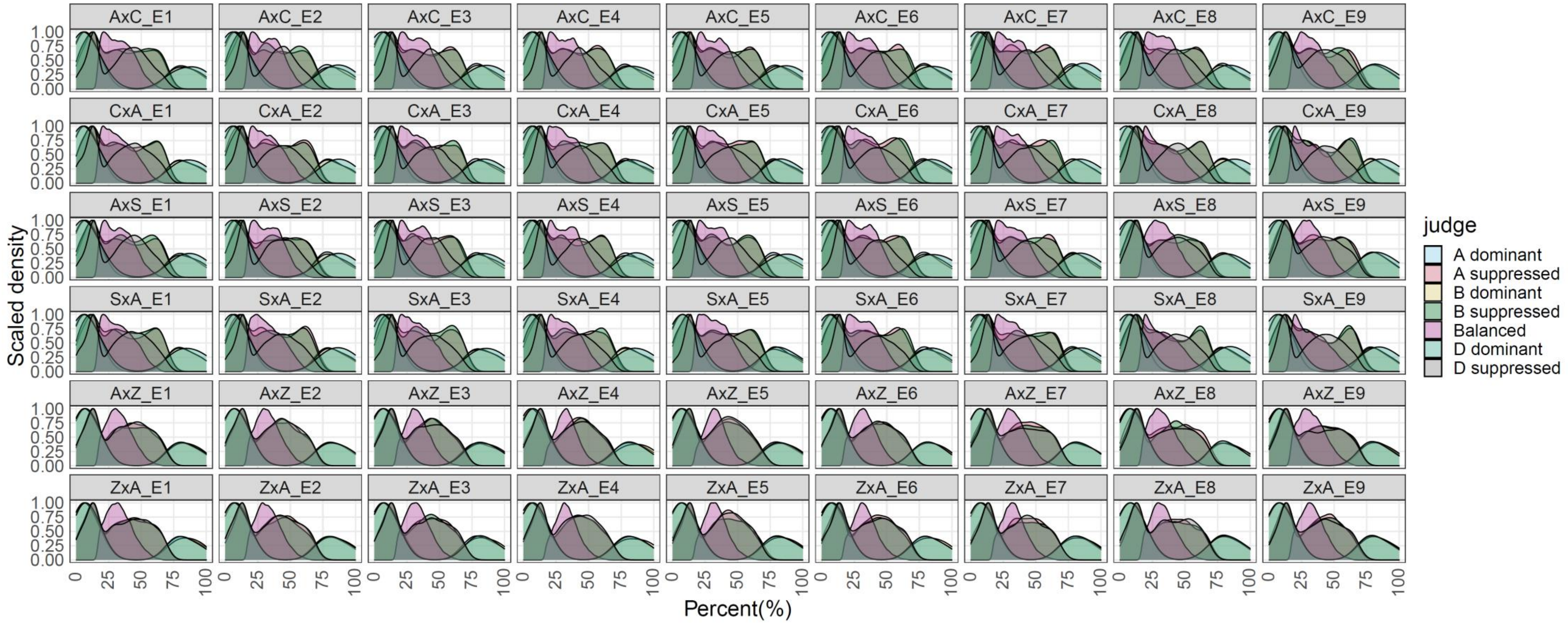

Supplementary Figure 24 Distribution of percent values for each homeologous gene in different categories. Triads were classified into seven categories based on the percent values. Density plots of percent values for all genes in each category are shown. Different categories are shown as different colors.

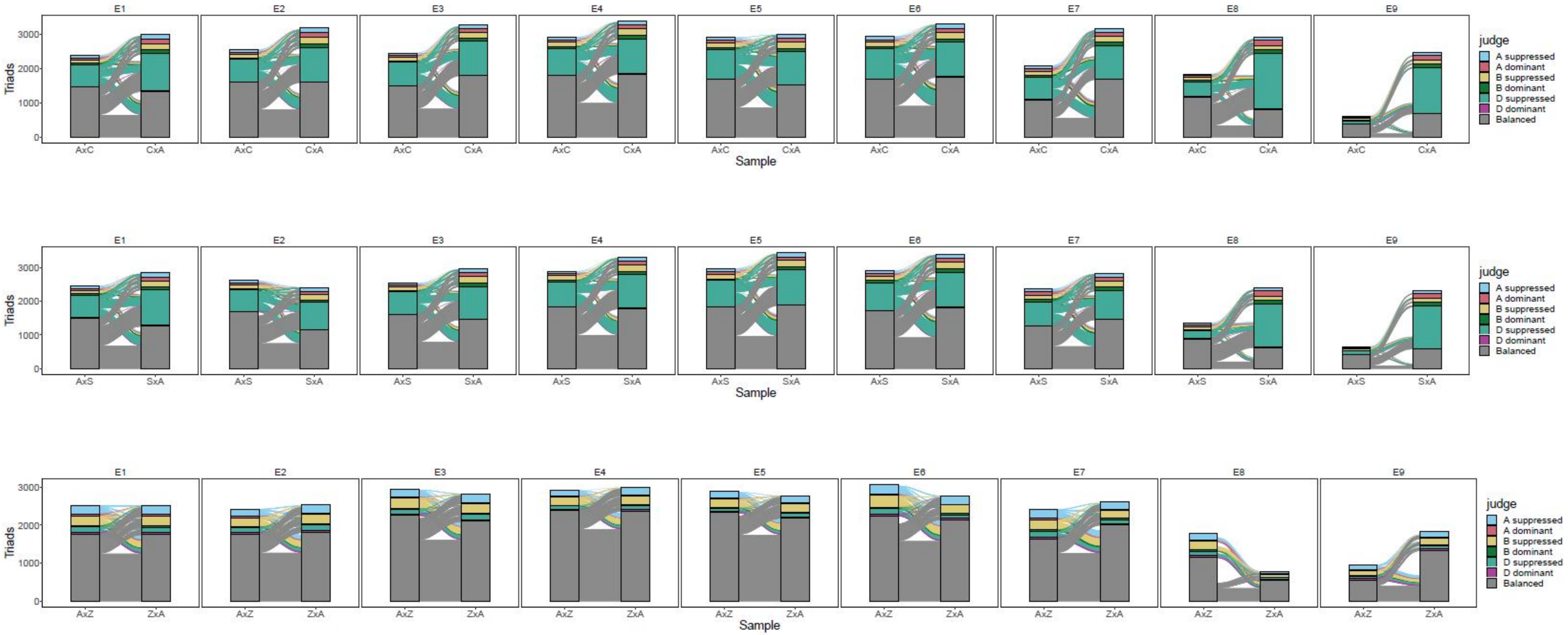

Supplementary Figure 25 Changes in triad classification between reciprocal crosses at each developmental stage. Triads were classified into seven categories based on their expression levels. Links between samples represent the triads that flow to the same as well as a different category between reciprocal cross. Triads belonging to each of the seven categories were colored to show shifts of triads between different categories.

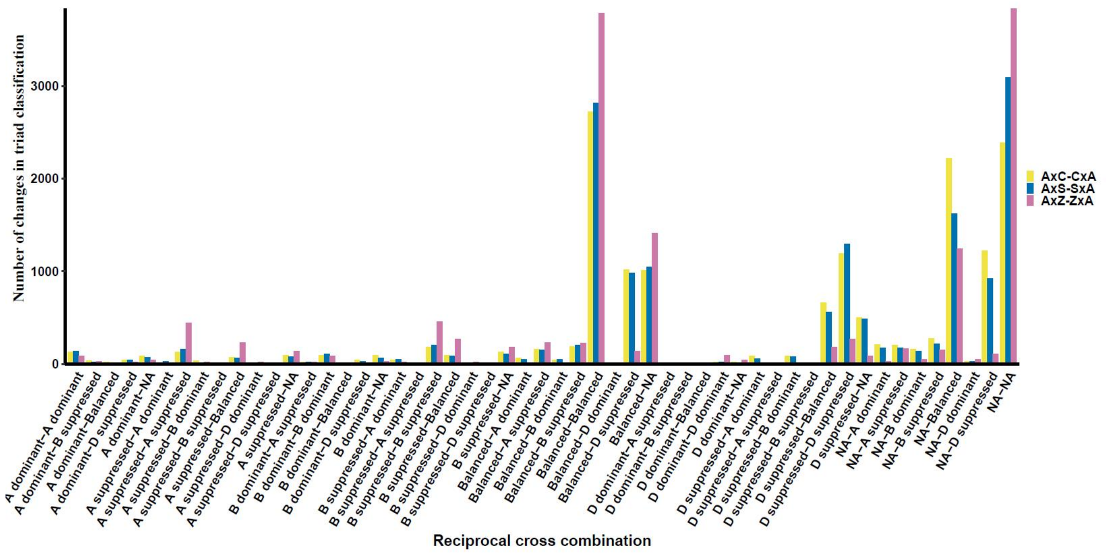

Supplementary Figure 26 Number of changes in triad classification between reciprocal crosses. Triads were classified into seven categories based on their expression levels including balanced, A dominant, B dominant, D dominant, A suppressed, B suppressed and D suppressed. NA, non-expressed. Number of changes between categories in three pairs of reciprocal crosses (AxC-CxA, AxS-SxA and AxZ-ZxA) were counted and plotted.

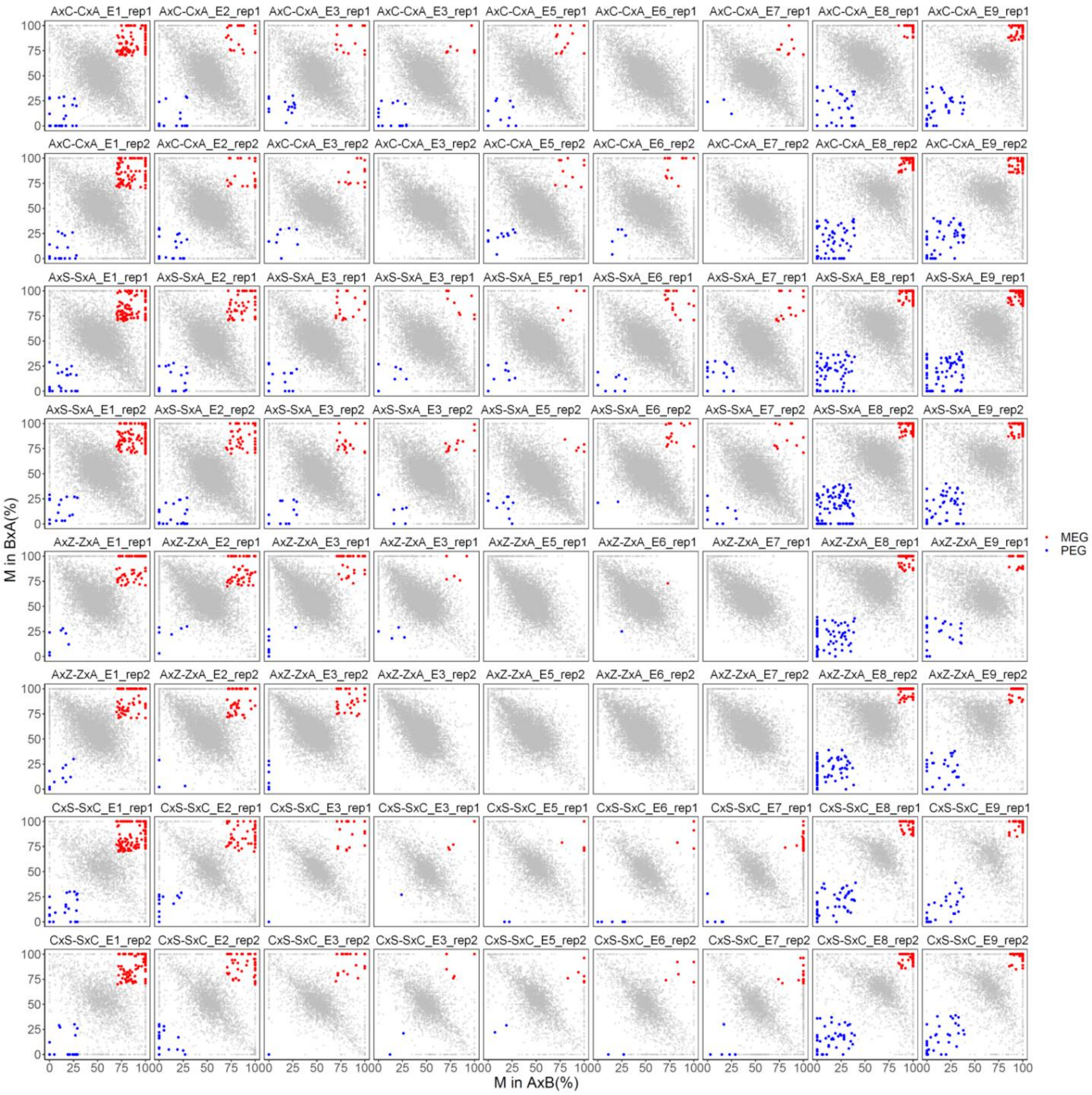

Supplementary Figure 27 Scatter plots for imprinting genes. Allele-specific expressionin each of the reciprocal cross pair at nine stages (E1-E9) in each of the two replicates (rep1 and rep2) were plotted. X axis (M in AxB (%)) represents maternal fraction of reads for genes from AxC, AxS, AxZ or CxS while y axis (M in BxA(%)) represents maternal fraction of reads for genes from BxA (CxA, SxA, ZxA and SxC), respectively. 100% is exclusively maternal and 0% is exclusively paternal.

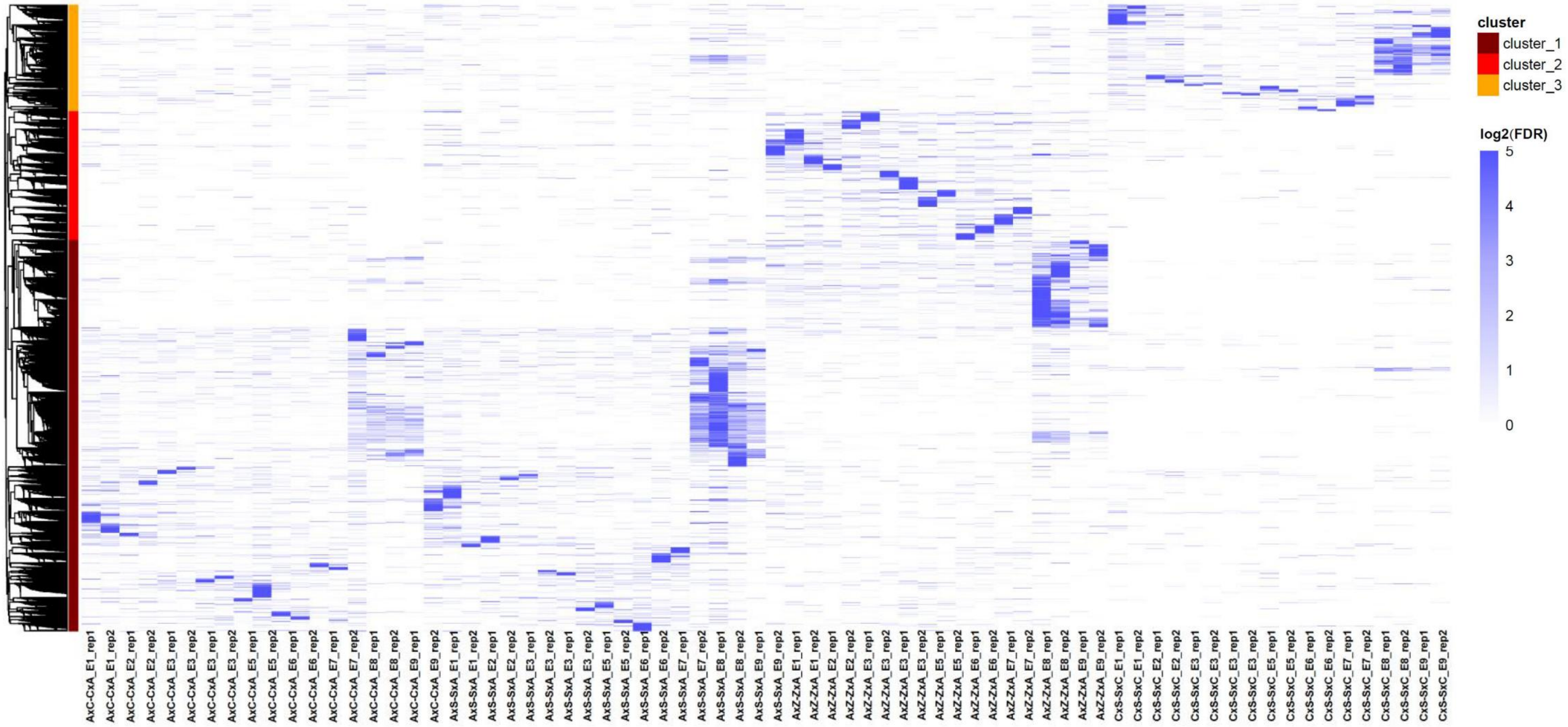

Supplementary Figure 28 Identification of imprinting genes. Allele-specific SNPs were identified and processed into allele-specific, per-gene counts (see details in Methods, Picard and Gehring, 2014). Paternally derived reads in two reciprocal

crosses of both biological replicates (>10 SNP-associated reads per cross) were counted. Chi square goodness-of-fit test was performed for each gene using paternally derived reads. Adjusted pvalues (FDR) were obtained for each gene. Heatmap show the log2-transformed FDR values of imprinting genes in four pairs of reciprocal crosses including AxC against CxA (AxC-CxA), AxS against SxA (AxS-SxA), AxZ against ZxA(AxZ-ZxA) and CxS against SxC(CxS-SxC).
