# Supplementary material for: Asymmetric gene expression in grain development of reciprocal crosses between tetraploid and hexaploid wheats
Source: Commun Biol. 2022 Dec 23;5:1412. doi: 10.1038/s42003-022-04374-w (PMC9789062; doi:10.1038/s42003-022-04374-w)
Supplement: Supplementary file 3 — Description of Additional Supplementary Data [file 42003_2022_4374_MOESM3_ESM.pdf]

**Description of Additional Supplementary Data Files**

**File name: Supplementary Data 1**

**Description:** Sample information in this study.

**File name: Supplementary Data 2**

**Description:** Mapping statistics of RNA sequencing data.

**File name: Supplementary Data 3**

**Description:** Genes identified in this study and gene annotation.

**File name: Supplementary Data 4**

**Description:** Differentially expressed genes between stages in embryos.

**File name: Supplementary Data 5**

**Description:** Differentially expressed genes between stages in embryo development pathways.

**File name: Supplementary Data 6**

**Description:** Differentially expressed genes between samples in embryos.

**File name: Supplementary Data 7**

**Description:** Differentially expressed genes between samples in embryo development pathways.

**File name: Supplementary Data 8**

**Description:** AS events identified in embryos.

**File name: Supplementary Data 9**

**Description:** Differential alternative splicing events between reciprocal pair.

**File name: Supplementary Data 10**

**Description:** Differentially expressed genes between samples involved in AS pathways.

**File name: Supplementary Data 11**

**Description:** Identification of homeologous genes in this study.

**File name: Supplementary Data 12**

**Description:** Triad classification changes between reciprocal crosses at each development stage.

**File name: Supplementary Data 13**

**Description:** GO enrichment analysis of triads showing different classifications between reciprocal cross.

**File name: Supplementary Data 14**

**Description:** SNP identification between reciprocal cross pairs.

**File name: Supplementary Data 15**

**Description:** Imprinting genes identified in this study.

**File name: Supplementary Data 16**
**Description:** Primers and probes used in this study.

**File name: Supplementary Data 17**

**Description:** Source data for Figure 2a, Figure 2b, Figure 3b, Figure 3f, Figure 3g, Figure 4a, Figure 4b, Figure 4d, Figure 4f, Figure 4g, Figure 5b, Figure 5d, Figure 6c, Figure 6e, Figure 7a, Figure 7b and Figure 7e.
